# Subclinical persistence of residual acral melanoma in situ after treatment with topical imiquimod and retinoid creams

**DOI:** 10.1016/j.jdcr.2023.12.017

**Published:** 2024-01-13

**Authors:** Jenne P. Ingrassia, Elizabeth Greenwald, Shane Meehan, Jennifer A. Stein, Tracey N. Liebman

**Affiliations:** a Ronald O. Perelman Department of Dermatology, New York University Grossman School of Medicine, New York, New York; b New York Medical College, School of Medicine, Valhalla, New York

**Keywords:** acral melanoma, imiquimod, skin cancer, surgical excision

## Introduction

Imiquimod is currently approved for use by the US Food and Drug Administration for the treatment of superficial basal cell carcinoma, actinic keratosis, and genital warts but has been increasingly utilized off-label in the management of lentigo maligna (LM) and cutaneous metastatic melanoma. There is a gap in the literature regarding the role of imiquimod in the management of acral lentiginous melanoma (ALM), though a few case reports suggest it may be an effective primary or adjuvant treatment option.^1^^,^^2^ Herein, we report a case of subclinical persistence of residual ALM in situ following adjuvant therapy with imiquimod and retinoid creams and highlight the limitations of using imiquimod therapy in the management of ALM.

## Case report

A 46-year-old woman with no significant medical history developed a rapidly enlarging pigmented lesion on the right plantar foot; a biopsy was performed and revealed invasive ALM with a depth of 2.25 mm. The patient underwent a radical excision. The pathology revealed residual ALM in situ close to the margins. A right inguinal sentinel lymphadenectomy was performed and did not show evidence of metastasis. Over the following 3 months, the patient underwent 2 additional surgical excisions with grafting, but the ALM persisted at the surgical margins following each surgery.

**Fig 1:**
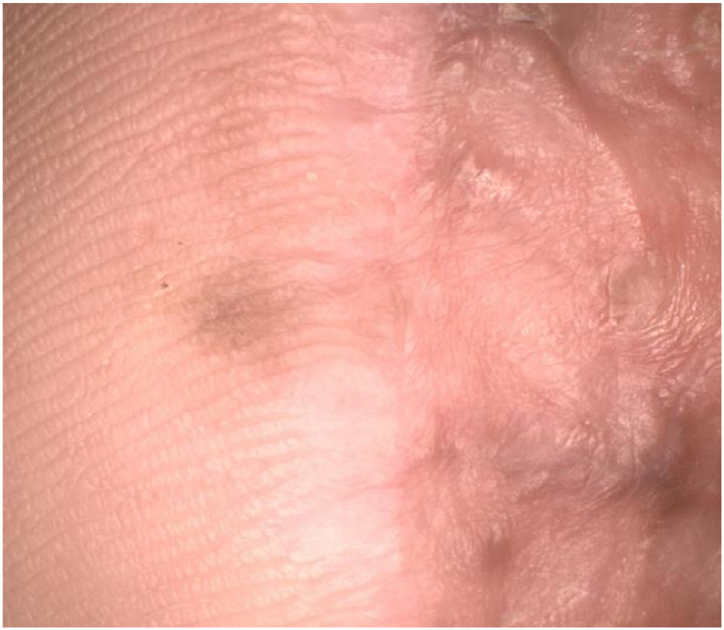
Clinical image of a *brown* pigmented macule medial to the surgical scar and graft site prior to treatment with imiquimod.

**Fig 2:**
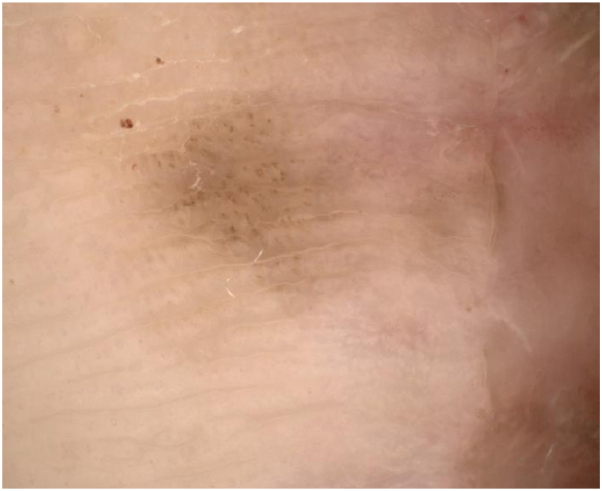
Dermoscopic image of pigmentation before treatment with topical imiquimod and tretinoin. Scattered globules are seen with homogenous *brown* pigmentation across ridges and furrows.

The patient was referred to dermatology to discuss nonsurgical management options given the persistent tumor margins despite repeated surgical intervention. On presentation, there was a 6-mm brown pigmented macule adjacent to the surgical scar and graft site (Fig 1), at the site of known residual melanoma in situ. Dermoscopy revealed homogenous brown pigmentation along the acral ridges and furrows with irregular dots and globules scattered throughout the lesion (Fig 2). The patient began treatment to the pigmented area with a combination topical therapy of 5% imiquimod and tretinoin 0.05% creams. This regimen was based on the findings of a clinical trial,^3^ which showed improved tumor clearance with a combination of imiquimod and tazarotene vs imiquimod alone in the treatment of LM. Our patient’s insurance did not cover tazarotene, which was initially prescribed. She was initially instructed to use imiquimod twice daily, 5 days a week, and tretinoin twice daily, 2 days a week, but there was no clinical response after several weeks. Thus, the patient was instructed to apply imiquimod twice daily and tretinoin once daily for 12 weeks.

**Fig 3:**
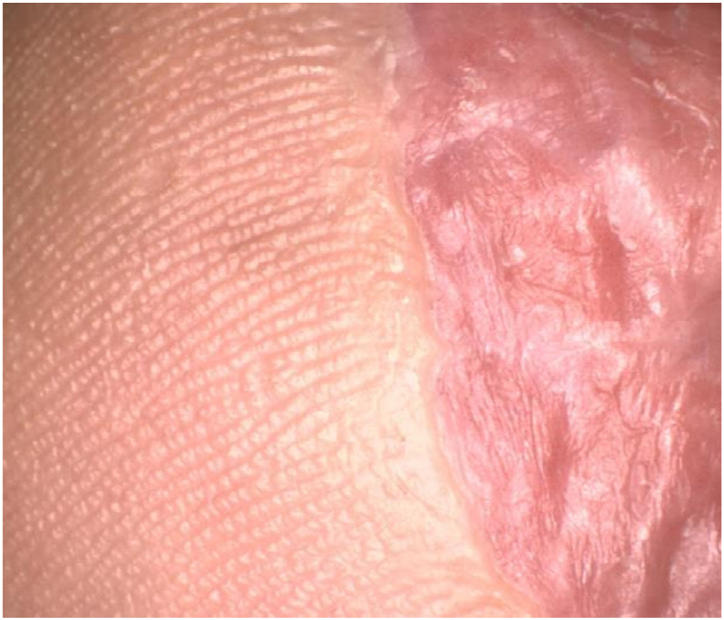
Clinical image of the affected site after 12 weeks of combination imiquimod and topical retinoid therapies showing near clinical clearance of the pigment.

**Fig 4:**
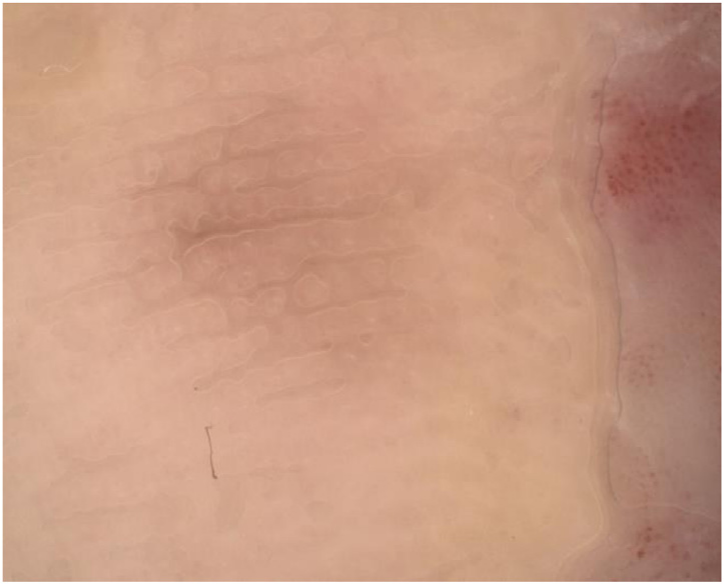
Dermoscopic image of pigmentation after treatment with topical imiquimod and tretinoin. Only faint homogenous residual pigment is evident after treatment.

After 6 weeks, dermoscopy showed significant fading of the lesion with few pigment globules. The graft site was red and irritated, so the regimen was decreased to 4 times per week, which was well tolerated. After 6 months, there was clinical and dermoscopic near clearance of the pigment (Figs 3 and 4). We performed a punch biopsy to determine if the remaining pigment represented dyschromia or tumor persistence and the histopathology revealed persistent ALM in situ. The patient underwent 2 additional staged excisions, and tumor-free margins were finally achieved. Unfortunately, the patient developed recurrence and progression of her disease 3 years later with metastasis to the lymph nodes, brain, and bladder. At the time of her recurrence, there was no evidence of a new primary melanoma or localized recurrence.

## Discussion

The standard treatment for ALM is wide local excision with tumor-free margins, but adequate margins may be difficult to achieve due to the extent of the disease and anatomic limitations. For tumors with increased potential for subclinical extension, like ALM, surgical techniques such as frozen section Mohs with immunohistochemical stains may improve margin control.^4^ When additional surgeries are not feasible, nonsurgical options like topical imiquimod may be used in an attempt to treat the residual disease. In this case, clear margins could not initially be obtained with multiple surgical excisions. The patient was treated with adjuvant imiquimod and tretinoin therapy to help clear the margins. Imiquimod was combined with a retinoid to improve the penetrance of the drug on the hyperkeratotic acral surface by disrupting the stratum corneum and decreasing the thickness of the skin barrier.^3^ After 6 months, the patient clinically improved, showing decreased pigmentation, but a biopsy of the area showed persistent melanoma in situ.

The literature is limited on the use of imiquimod in the management of ALM, though existing case reports suggest it may be an effective treatment.^1^^,^^2^ In 1 case report,^1^ a patient with ALM in situ declined surgery and was instead treated with 5% imiquimod for weeks.^1^ The patient appeared clinically clear and there was no evidence of systemic disease after 8 months. However, a repeat biopsy was not performed and the follow-up time reported was limited. Therefore, it is unclear if the imiquimod successfully treated the melanoma. Another small case series^2^ of 2 patients, 1 with invasive ALM and 1 with ALM in situ, used adjuvant imiquimod therapy to treat melanoma in situ following surgical excision. The patients were treated for 8 weeks and repeat biopsies did not show histopathologic evidence of residual disease.^2^

Unlike prior reports,^1^^,^^2^ our findings demonstrate that imiquimod was not an effective therapy for our patient. A biopsy was required to detect disease persistence, given that the lesion had clinically faded and appeared markedly improved. A retrospective analysis of 40 patients treated with imiquimod for LM on the face similarly found that 3 patients responded clinically, but subsequent punch biopsies revealed histopathologic disease.^5^ Furthermore, a clinical trial found tumor persistence of LM upon biopsy in a high percentage of cases after treatment with imiquimod and tazarotene creams.^3^ Taken together, the results of prior studies and our results suggest that subclinical disease may persist after treatment with imiquimod, even if the affected area appears to be clinically cleared.

This case highlights the limitations of using imiquimod therapy in the management of ALM. If imiquimod therapy is utilized in the management of ALM, we recommend performing a biopsy after treatment even if the area appears clinically clear. While larger, prospective studies are needed to better understand the role of imiquimod in the management of ALM, our case serves as a warning that imiquimod may not only fail to treat ALM, but it can also mask clinical and dermoscopic evidence of residual disease.

## Conflicts of interest

Dr Liebman and Dr Stein have diagnosed patients for MoleSafe, USA, for which she has received payments from the NYU Langone Department of Dermatology. Author Ingrassia, Dr Greenwald, and Dr Meehan have no conflicts of interest to declare.
